# Diagnostic and predictive ability of hyperbilirubinemia severity in cats: A multicenter retrospective study

**DOI:** 10.1111/jvim.17005

**Published:** 2024-02-15

**Authors:** Xavier Salord Torres, Kamalan Jeevaratnam, Imogen Schofield, Samantha Taylor, Jennifer Stallwood, Menai Heyes, Daniel Hughes, Pieter Defauw

**Affiliations:** ^1^ Lumbry Park Veterinary Specialists (CVS) Alton UK; ^2^ University of Surrey School of Veterinary Medicine Guildford UK; ^3^ CVS Group CVS House Diss UK; ^4^ Linnaeous Veterinary Limited Shirley UK; ^5^ International Society of Feline Medicine Tisbury UK; ^6^ Bristol Veterinary Specialists (former Highcroft Veterinary Referrals) (CVS) Bristol UK; ^7^ ChesterGates Veterinary Specialists (CVS) Chester UK

**Keywords:** biliary, bilirubin, icterus, jaundice, liver, obstruction

## Abstract

**Background:**

Total serum bilirubin concentration (TBIL) can provide useful information on several pathophysiological conditions in cats. Nevertheless, whether the variable severity classification of hyperbilirubinemia can reliably indicate certain disease processes or predict a biliary obstruction (BO) has not been investigated.

**Hypothesis/Objective:**

Determine if hyperbilirubinemia of variable severity can assist clinicians to identify BO, which often is considered a surgical emergency.

**Animals:**

Two‐hundred sixteen client‐owned cats.

**Methods:**

Data were retrospectively collected from all cats (January 2015‐August 2022) with an increased TBIL (>0.58 mg/dL [>10 μmol/L]) presented to 3 referral centers in the United Kingdom (UK). Presenting clinical features and diagnostic outcomes were collected. The predictive ability of TBIL to indicate BO was evaluated by multivariable binary logistic regression modeling and receiver operating characteristic (ROC) curves.

**Results:**

Median TBIL was 1.73 mg/dL (range, 0.59‐26.15; 29.5 μmol/L; range, 10.1‐447.1) with severity classification of hyperbilirubinemia categorized as mild (>0.58‐2.92 mg/dL; >10‐50 μmol/L; 68.1%), moderate (>2.92‐5.85 mg/dL; >50‐100 μmol/L; 17.6%), severe (>5.85‐11.70 mg/dL; >100‐200 μmol/L; 9.7%) and very severe (>11.70 mg/dL; >200 μmol/L; 4.6%). Biliary obstruction was present in 17 (7.9%) cats, all of which received recommendation for emergency surgery. Median TBIL in cats with BO (9.69 mg/dL; 165.7 μmol/L) differed significantly from those without obstruction (1.51 mg/dL; 25.8 μmol/L; *P* < .01). The optimal TBIL cut‐off to discriminate between cats with and without BO was ≥3.86 mg/dL (≥66 μmol/L) with a sensitivity of 94.1% and specificity of 82.4%. Using multivariable logistic regression, as age increased, the odds of BO increased significantly (odds ratio, 1.20; 95% confidence interval, 1.01‐1.42; *P* = .04).

**Conclusions and Clinical Importance:**

As part of a thorough clinical assessment, the severity classification of hyperbilirubinemia has the potential to predict the likelihood of a BO and to discriminate between cats that may or may not require surgery for BO at a suggested cut‐off of ≥3.86 mg/dL (≥66 μmol/L). Alongside TBIL, age is also useful when assessing for the likelihood of BO in a cat presented with hyperbilirubinemia.

**Abbreviations**
ALP: alkaline phosphatase
ALT: alanine transaminase
BO: biliary obstruction
FIP: feline infectious peritonitis
GGT: gamma‐glutamyl transferase
IQR: interquartile range
LRT: likelihood ratio test.
RBC: red blood cell count
TBIL: total serum bilirubin concentration
WBC: white blood cell count

## 1 INTRODUCTION

Total serum bilirubin concentration (TBIL) is a useful biochemical variable that, as part of a thorough clinical assessment, can provide information on several physiological functions and pathologic processes. Cats with hyperbilirubinemia often are presented with clinical icterus (jaundice) that may be identified on clinical examination. The principal causes of hyperbilirubinemia can be separated into 3 disease processes,^1^, ^2^, ^3^ which can be applied to cats: hemolysis (prehepatic), derangement of hepatocyte function to process and excrete bilirubin (hepatic), and obstruction of bile excretion into the duodenum (posthepatic).

In cases of systemic inflammation, such as sepsis or feline infectious peritonitis (FIP), hyperbilirubinemia can be present without evidence of hemolysis, liver disease, or cholestasis,^4^ and its cause and subsequent classification into a specific disease process (prehepatic, hepatic, posthepatic) is therefore less clear. It is speculated that bilirubin metabolism and excretion into the biliary system is compromised in cats with sepsis and FIP,^4^, ^5^ although FIP also has been reported to be associated with immune‐mediated hemolytic anemia.^6^

The underlying cause of hyperbilirubinemia in cats is not always evident, even after thorough diagnostic investigations. Availability and experience with hepatobiliary surgery and certain diagnostic modalities such as abdominal ultrasonography in primary practice may be limited. Therefore, assessment of TBIL alone may have the potential to influence clinical decisions in this setting. Relevant literature in the field of feline medicine^1^ describes different causes of clinical jaundice and categorizes them using different TBIL ranges. Most notably, a previous study reported that when TBIL is >11.7 mg/dL (200 μmol/L), the hyperbilirubinemia is associated with hepatic lipidosis or posthepatic obstruction, the latter often indicating a surgical emergency, and when TBIL is <5.85 mg/dL (100 μmol/L) then hemolysis, FIP, pancreatitis, amyloidosis and sepsis are more likely.^1^ However, despite the usefulness of such indicative ranges to guide veterinarians, these cut‐offs were, as far as the authors are aware, suggested based on expert opinion, whereas evidence‐based research is lacking. In humans, the degree of hyperbilirubinemia in isolation has been shown to be indicative of underlying obstructive clinical jaundice, helping differentiate benign and malignant causes.^7^

Although abdominal ultrasonography is a reliable imaging modality for differentiating extrahepatic obstruction and intrahepatic disease,^8^, ^9^, ^10^ not all primary care practices have the equipment and expertise to perform the required detailed examination using this imaging modality. Therefore, developing further the clinical utility of hyperbilirubinemia through a severity classification may assist the general practitioner in clinical decision‐making, such as the need for emergency referral, advanced imaging and potentially surgery. We sought to establish whether variable severity classification of hyperbilirubinemia could help identify underlying causes of hyperbilirubinemia, with particular interest in those conditions resulting in BO, which often is considered a surgical emergency. We hypothesized that hyperbilirubinemia of variable severity may be indicative of the underlying pathology causing it, especially causes resulting in BO.

## 2 MATERIALS AND METHODS

### 2.1 Study design

In this multicenter retrospective study, medical records of all cats with hyperbilirubinemia (TBIL above the upper limit of the laboratory reference range [10 μmol/L]) presented to 3 UK referral hospitals between January 2015 and August 2022 were reviewed. The CVS Group Ethics Committee approved this study (#CVS‐2022‐018). Data collected included date of presentation, signalment, presenting clinical features, investigation findings including abdominal imaging when available, and clinical outcome when available. Cats that had no abdominal imaging performed (eg, ultrasonography, radiography, computed tomography) were not excluded if diagnostic imaging was not deemed necessary to reach a diagnosis by the attending clinician. In cases in which TBIL was evaluated serially for example, daily measurements in hospitalized patients, only the first measurement was taken into account. Duplicated data and cases with incomplete patient data were excluded.

### 2.2 Total serum bilirubin concentration

The TBIL measurements were available from 3 different UK laboratories, all using the same upper limit for normal TBIL (0.58 mg/dL). For TBIL measurement, the following chemistry analyzers were used: Beckman Coulter AU680 (Beckman Coulter, Cassina, De'Pecchi, Italy) and Fujifilm Dri‐Chem NX500 (Fujifilm Corporation, Tokyo, Japan).

The severity classification of hyperbilirubinemia was established based on the following TBIL ranges: mild (>0.58‐2.92 mg/dL), moderate (>2.92‐5.85 mg/dL), severe (>5.85‐11.70 mg/dL) and very severe (>11.70 mg/dL). These ranges were determined by the authors, but previously described ranges were reviewed and used as guidance.^1^

### 2.3 Causes of hyperbilirubinemia

Taking into account the clinical diagnosis documented by the attending veterinarian and clinical history, medical records were reviewed retrospectively on a case‐by‐case basis to accurately assign a cause of hyperbilirubinemia to each case. Cases in which the cause of hyperbilirubinemia was not clear were classified as “nonattributable cause,” but diagnostic findings and concurrent morbidities still were recorded for each individual in the event that possible associations to explain the hyperbilirubinemia would exist. Diagnoses also were subclassified depending on whether the disorder leading to hyperbilirubinemia was prehepatic, hepatic or posthepatic. Assignment of each case to each category also was made by the authors, but consistent with relevant veterinary literature.^1^, ^2^, ^8^

Another subcategory also was created for all cases with documented BO, defined as an obstruction of the biliary system leading to impaired flow of bile from the liver to the intestinal tract.^11^ The diagnosis of BO was made based on abdominal ultrasonography, which is the accepted imaging modality for differentiating extrahepatic obstruction and intrahepatic (parenchymal) liver disease.^8^

### 2.4 Treatment recommendation and outcome

Treatment recommendations including the need for hepatobiliary surgery were recorded for all cases, including cats with a diagnosis of BO on diagnostic imaging. For the purposes of the study, recording “treatment recommendation” over “treatment performed” was considered the ideal way to document the treatment that was deemed clinically necessary for each patient. Final outcome, including death and euthanasia, was recorded when available.

### 2.5 Statistical analysis

Analyses were performed using a computer software package, Stata 17.0 (Stata Corps, TX, USA). Continuous data were assessed for normality graphically and using Shapiro‐Wilk tests. Mean and SD were reported for normally distributed data. Median, interquartile range (IQR) and range were reported for nonnormally distributed data. Comparison of categorical variables, presented by showing the count and percentage, were made using chi‐squared tests, whereas Fisher's exact tests were used for variables with <5 observations in a category. Comparisons of continuous risk factors variables were made using nonparametric tests: Wilcoxon Rank sum tests and Kruskal‐Wallis tests.

The discriminatory ability of TBIL and identification of optimal cut‐offs to predict BO were assessed using receiver operating (ROC) curves. This ability was further evaluated by multivariable binary logistic regression modeling. Variables considered a priori as possible confounding factors, and assessed within the multivariable model included age, sex, serum albumin concentration, white blood cell count (WBC), neutrophil count, alkaline phosphatase (ALP) activity, alanine transaminase (ALT) activity, gamma‐glutamyl transferase (GGT) activity, red blood cell count (RBC) and hemoglobin concentration. Absolute values of laboratory variables were assessed within the model. Hospital site was included in the model as a fixed effect to account for any clustering within the models, and nonnormally distributed data were log‐transformed for analysis. Continuous variables with a marked right skew were logarithmically transformed for regression analysis. Risk factors that had a broad association with BO during univariable analysis (likelihood ratio test [LRT] = *P* < .2) were considered for multivariable evaluation. Multivariable model‐building used a backwards stepwise manual approach. Confounding effects were assessed by observing for at least a 10% change in the odds ratios (OR) after inclusion of an additional risk factor. Continuous variables were assessed for linearity with the outcome by comparing categorical and linear nested models of the variable, using the LRT. Variables were considered collinear if correlation coefficients (rho) were >0.8. Biologically plausible pairwise interactions in the final model were examined using the LRT. Goodness‐of‐fit and discrimination of the final model were evaluated using the Hosmer‐Lemeshow test and the area under the ROC curve (AUROC). Significance was set at *P* < .05 throughout.

## 3 RESULTS

### 3.1 Cats

Two‐hundred sixteen (216) cats with hyperbilirubinemia were included in the study of which 139 (64.3%) were males (90.66% neutered, 9.36% intact) and 77 (35.6%) were females (83.1% spayed, 16.9% intact). The mean age was 7.9  ±4.6 years. Represented cat breeds included Domestic Short Hair (n = 131), Domestic Long Hair (15), British Short Hair (15), and other breeds (58) which included Main Coon (8), Bengal (7), Ragdoll (7), Burmese (5), Persian (4), Tonkinese (4), Birman (3), Oriental (2), Savannah (2), Siamese (2), Siberian Forest (2), Balinese (1), Exotic Short Hair (1), Havana Brown (1), Munchkin (1), Norwegian Forest (1), and 3 other crossbreed cats.

### 3.2 Causes of hyperbilirubinemia

Median TBIL was 1.73 mg/dL (range, 0.59‐26.15; IQR, 0.9‐3.57). Hyperbilirubinemia was classified as mild (n = 147; 68.1%), moderate (38; 17.6%), severe (21; 9.7%) or very severe (10; 4.6%). Abdominal imaging was performed in 185 cases (ultrasonography [n = 170], computed tomography [n = 14], radiography [n = 1]). The most common attributable causes of hyperbilirubinemia were biliary tree disease (55; 25.5%), hepatic parenchymal disease (23; 10.7%), and FIP (17; 8.3%). A significant difference in median TBIL (*P* < .001) was found across the 7 attributable causes (Table 1). Additionally, in 80 cases (37%) the cause of hyperbilirubinemia was not clear or proven. Median TBIL concentration was 1.2 mg/dL (IQR, 0.85‐2.09) in the 80 cases with no attributable cause. Table 2 summarizes the most common diagnoses and comorbidities found in those cases with nonattributable hyperbilirubinemia.

**TABLE 1:** Median bilirubin concentrations for different attributable causes of hyperbilirubinemia in 216 cats.

	Attributable cause of hyperbilirubinemia			Median total serum bilirubin concentration (mg/dL; μmol/L)	Interquartile range (range) (mg/dL; μmol/L)
	Number (n)		%		
Prehepatic	Feline infectious peritonitis (18)		8.3	1.44; 24.7	1.13‐3.22 (0.79‐5.04); 19.4‐55.0 (13.5‐86.1)
	Hemolysis (14)		6.5	0.92; 15.8	0.73‐2.16 (0.64‐9.94); 12.5‐37.0 (11.0‐170.0)
	Sepsis (9)		4.2	1.13; 19.3	0.82‐1.34 (0.76‐5.91); 14.0‐22.9 (13.0‐101.0)
Hepatic	Hepatic parenchymal disease (23)	Primary/secondary hepatic neoplasia (13); hepatic lipidosis (5); hepatitis/hepatopathy (3^a^); congenital hepatic fibrosis/ductal plate malformation (2)	10.7	3.42; 58.4	0.88‐7.08 (0.64‐21.12); 15.0‐121.0 (10.9‐361.1)
	Biliary tree disease (55)	Cholangitis/cholangiopathy (41)	25.4	2.81; 48.0	1.37‐8.36 (0.59‐26.15); 23.4‐143.0 (10.1‐447.1)
Posthepatic		Biliary mass (7); cholelithiasis (6); diaphragmatic hernia causing biliary obstruction (1)			
	Pancreatic disease (17)	Pancreatitis/pancreatopathy (13); pancreatic mass (4)	7.9	1.54; 26.3	1.23‐2.40 (0.61‐14.79); 21.0‐41.0 (10.5‐252.9)
	Nonattributable cause (80) See Table 2		37.0	1.20; 20.5	0.85‐2.09 (0.60‐7.53); 14.5‐35.8 (10.2‐128.7)

^a^ This includes a case of chronic pyogranulomatous hepatitis caused by Yersinia pseudotuberculosis.

**TABLE 2:** Most common diagnoses and comorbidities in 216 cats with hyperbilirubinemia with no clear cause.^a^

Diagnosis/comorbidity	Number of cats
Polytrauma/recent history of trauma (without evidence of biliary tree involvement documented on abdominal imaging)	17
Kidney disease (acute or chronic)	15
Enteropathy	9
Cardiac disease	7
Diabetes mellitus/diabetic ketoacidosis	4
Abdominal mass (without biliary tree involvement)	3
Lower airway disease	3
Lymphadenopathy	3
Ocular disease	3

^a^ Note some cats had more than one disease/comorbidity.

**FIGURE 1:**
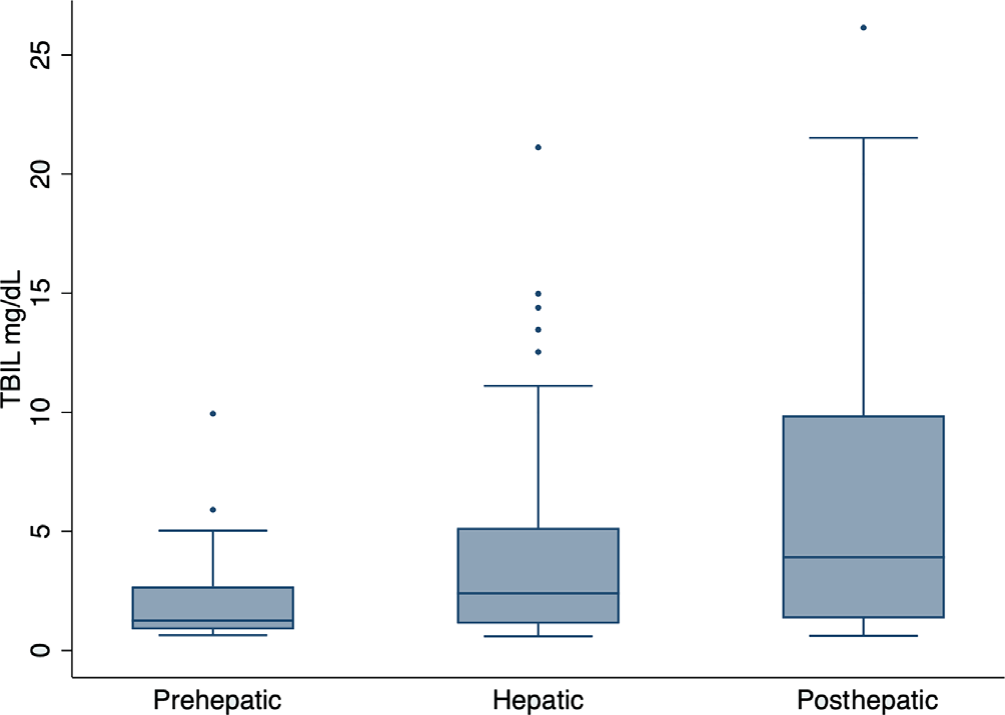
Box and whisker plot demonstrating the difference in median total serum bilirubin for 216 cats with prehepatic, hepatic and posthepatic causes of hyperbilirubinemia.

Median TBIL was 1.26 mg/dL (range, 0.64‐9.94; IQR, 0.88‐2.7) for the 41 cats with prehepatic cause of hyperbilirubinemia, 2.4 mg/dL (range, 0.59‐21.12; IQR, 1.12‐5.15) for the 64 cats with hepatic causes and 3.92 mg/dL (range, 0.61‐26.15; IQR, 1.34‐9.88) for the 31 cats with posthepatic causes (Figure 1). Possible cut‐offs to discriminate among pre, hepatic and posthepatic causes of hyperbilirubinemia were explored but were poorly sensitive and specific because of overlap among groups.

### 3.3 Biliary obstruction

**FIGURE 2:**
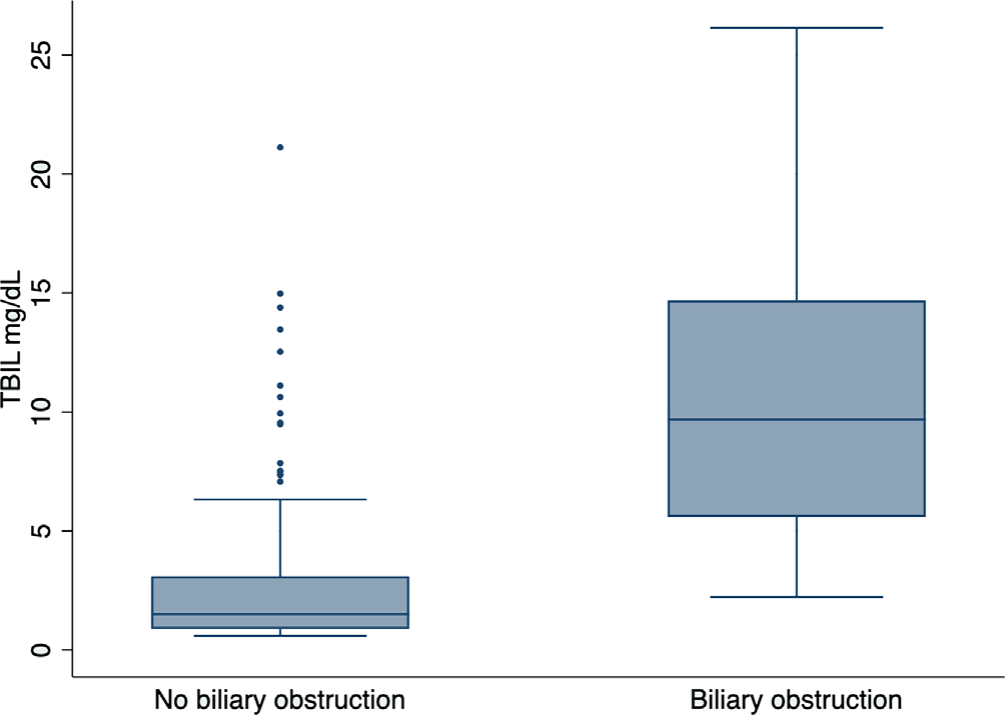
Box and whisker plot demonstrating the difference in median total serum bilirubin concentration (TBIL) for 216 cats with and without biliary obstruction. Boxes represent the interquartile range from the 25th and 75th percentile. The horizontal bar in each box represents the medial value. The whiskers indicate the range of data values unless outliers are present, in which case the whiskers extend to a maximum of 1.5 times the interquartile range. Such outlying data points are represented by dots.

Biliary obstruction diagnosed by abdominal ultrasonography was present in 17 (7.9%) cats with hyperbilirubinemia. Median bilirubin concentration in those with BO was 9.69 mg/dL (range, 2.22‐26.15; IQR, 5.58‐14.68) which was significantly higher compared with those without BO (1.51 mg/dL [range, 0.59‐21.12; IQR, 0.88‐3.1;]; *P* < .001). A significant strong positive association was found between TBIL and presence of BO (*P* < .001; Figure 2). Hyperbilirubinemia of increasing severity classification had far greater odds of BO (Table 3); therefore, the more severe the hyperbilirubinemia, the greater the odds of BO.

**TABLE 3:** Categorization of hyperbilirubinemia categories with the subsequent diagnosis of biliary obstruction in 216 cats.

Hyperbilirubinemia category (mg/dL; μmol/L)	Biliary obstruction (%)	No biliary obstruction (%)	Odds ratio (95% CI)
Mild (>0.58‐2.92 mg/dL; >10‐50 μmol/L)	1 (5.9)	146 (73.4)	Baseline
Moderate (>2.92‐5.85 mg/dL; >50‐100 μmol/L)	4 (23.5)	34 (17.1)	17.2 (1.86‐158.6)
Severe [>5.85‐11.70 mg/dL; >100‐200 μmol/L)	7 (41.2)	14 (7.0)	73.0 (8.37‐636.7)
Very severe (>11.70 mg/dL; >200 μmol/L)	5 (29.4)	5 (2.5)	146.0 (14.3‐1492.7)

All 17 cats with BO received the recommendation by the treating clinician to undergo urgent hepatobiliary surgery. Therefore, hyperbilirubinemia of increasing severity was strongly associated with greater odds of surgical intervention as a treatment recommendation. In other words, using the data collected in our study, the bilirubin concentration was highly discriminatory for cats requiring surgical intervention or not (AUROC, 0.92; 95% CI, 0.88‐0.97). Of the 17 cats in which surgery was recommended, 5 underwent surgery (2 were euthanized shortly after surgery, and follow‐up was lost for the other 3) and 12 cats did not undergo surgery for different reasons (7 of these cats were euthanized after diagnosis, and follow‐up was lost for the other 5 cats).

The mean age of cats with BO was 10.1 years (range, 2.25‐18.2 years); cats with a mass lesion involving the biliary tree had a similar mean age (10.5 years; range, 5.25‐16.5 years).

#### 3.3.1 Predictive ability of total bilirubin concentration to identify biliary obstruction

**FIGURE 3:**
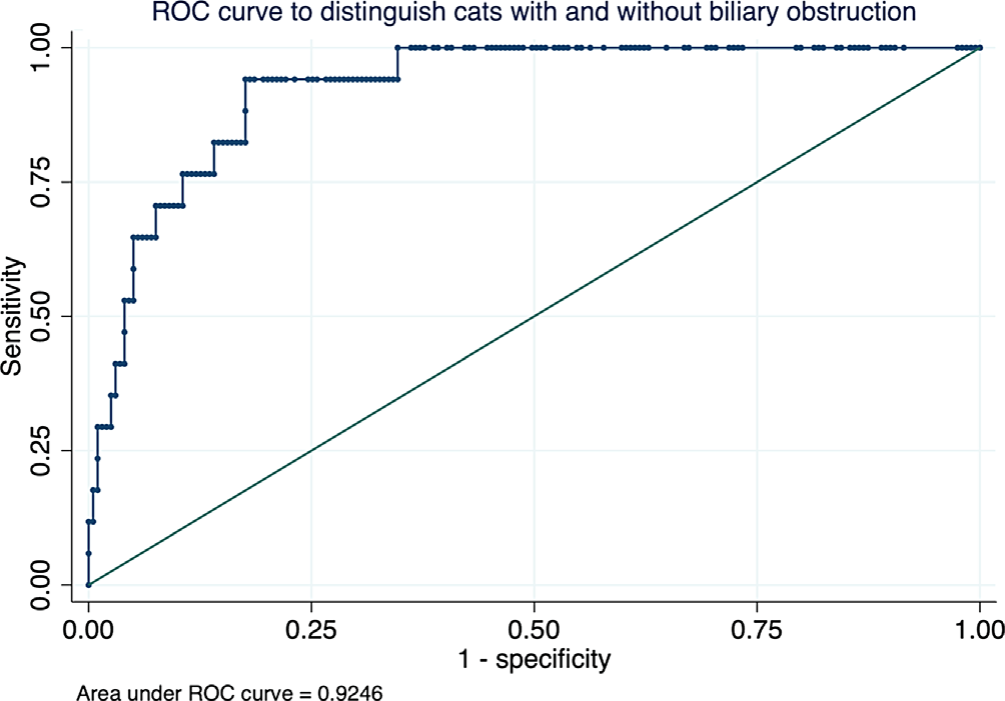
Receiver operating characteristic (ROC) curve discriminating between cats with and without biliary obstruction in 216 cats with hyperbilirubinemia.

Total serum bilirubin concentration was highly discriminatory for cats with BO (AUROC, 0.92; 95% CI, 0.88‐0.97). The optimal TBIL cut‐off to discriminate between cats with and without BO was ≥3.86 mg/dL, which had a sensitivity of 94.1% and a specificity of 82.4% (Figure 3).

Using the previously described cut‐offs to discriminate among different causes of hyperbilirubinemia^1^ with a suggested total bilirubin cut‐off ≥5.85 mg/dL, the sensitivity for BO was 70.6% and specificity was 90.5%. At a suggested cut‐off of ≥11.7 mg/dL, the sensitivity for BO was 35.3% and specificity was 97.5%.

#### 3.3.2 Clinical factors associated with biliary obstruction

The association between TBIL and BO was further assessed using multivariable logistic regression modeling. Log_10_ (TBIL), age, sex, albumin, log_10_ (ALP), log_10_ (ALT), log_10_ (GGT), and log_10_ (RBC) were at least weakly associated with the presence of BO (Table 4) and were taken forward to multivariable analysis. The final multivariable model only retained log_10_ (TBIL) and age (Table 5), meaning that both variables were independently and linearly associated with BO. As log_10_ (TBIL) increased, the odds of BO increased (OR, 9.58; 95% CI, 3.95‐23.23; *P* < .001). As age increased, the odds of BO also increased significantly (OR, 1.20; 95% CI, 1.01‐1.42; *P* = .04). Hospital site was retained a priori within the analysis throughout to account for any clustering in the model. No association was found between hospital site and presence of BO (*P* = .43) or log_10_ (TBIL; *P* = .47).

**TABLE 4:** Descriptive statistics and univariable logistic regression of risk factors for biliary obstruction in 216 cats with hyperbilirubinemia, after accounting for hospital attended.

Variable	Obstruction (SD/%)	No obstruction (SD/%)	OR	95% CI	*P*‐value
Log_10_ (total bilirubin)	5.0 (0.7)	3.4 (0.8)	8.85	3.79‐20.65	<.001
Age (years)	10.1 (4.5)	7.7 (4.6)	1.13	1.00‐1.26	.04
Sex (female)	9 (11.7)	68 (88.3)	Baseline		
Sex (male)	8 (5.8)	131 (94.2)	0.46	0.17‐1.25	.13
Albumin	31.8 (5.6)	25.3 (6.1)	1.20	1.09‐1.33	<.001
Log_10_ (white blood cell count)	2.5 (0.5)	2.4 (0.8)	1.22	0.60‐2.48	.59
Log_10_ (neutrophil count)	2.3 (0.6)	2.0 (1.0)	1.33	0.72‐2.45	.37
Log_10_ (ALP)	5.1 (0.9)	3.6 (1.2)	2.73	1.67‐4.46	<.001
Log_10_ (ALT)	6.6 (0.4)	4.9 (1.2)	7.08	2.59‐19.34	<.001
Log_10_ (GGT)	2.8 (1.0)	1.4 (0.9)	4.56	2.34‐9.31	<.001
Log_10_ (red blood cell count)	2.0 (0.2)	1.7 (0.4)	9.18	1.34‐62.98	.02

**TABLE 5:** Multivariable binary logistic regression model to assess the risk factors for biliary obstruction in 216 cats with hyperbilirubinemia.

Variable^a^	OR	95% CI	*P*‐value
Log_10_ (total bilirubin)	9.58	3.95‐23.23	<.001
Age (years)	1.20	1.01‐1.42	.04

^a^ The following variables were evaluated and eliminated during multivariable binary logistic regression modeling; sex, albumin, Log (ALP), Log (ALT), Log (GGT) and Log (RBC). Hospital site was retained a priori within the analysis as a fixed effect to account for any clustering in the model (*P* = .47).

This final multivariable model had excellent discrimination for BO (AUROC, 0.93; 95% CI, 0.88‐0.97) and model fit (Hosmer Lemeshow *P* = .31).

## 4 DISCUSSION

In the clinical setting of a cat presented with hyperbilirubinemia, bilirubin concentrations have the potential to influence investigation and treatment considerations. In our study, the degree of hyperbilirubinemia had high potential to discriminate between cats with and without BO. Median TBIL in those cats with BO was 9.69 mg/dL and differed significantly from those without BO (1.51 mg/dL). Therefore, cats with severe and very severe hyperbilirubinemia classification had far greater odds of BO than those with mild and moderate hyperbilirubinemia. This pattern of increase in TBIL can assist veterinarians to assess whether a cat is more or less likely to need emergency hepatobiliary surgery, and for which referral to a specialist for abdominal imaging and potential surgery may be indicated. We evaluated whether TBIL can differentiate among the different causes of hyperbilirubinemia, with particular interest in those cases considered to be a surgical emergency. In humans with obstructive jaundice caused by biliary disease, bilirubin concentrations in isolation have been shown to represent an important tool for discriminating among different underlying causes,^7^ but such a finding has not been reported in small animal medicine.

Abdominal ultrasonography has been considered critical in identifying whether extrahepatic biliary duct obstruction is present when TBIL is >5.85 mg/dL.^1^ However, when this cut‐off was applied to our data, the sensitivity for identifying cats with BO (and therefore cats possibly requiring hepatobiliary surgery) was only 70.6% with a specificity of 90.5%. At the suggested cut‐off of >11.7 mg/dL, the sensitivity to detect cats with BO was even lower at 35.3%, with a specificity of 97.5%. Therefore, this higher cut‐off (proposed by the *British Small Animal Veterinary Association Manual of Feline Practice 2013*) would correctly identify most cats that will not require surgery, showing good ability to identify nonsurgical cases, but would miss up to 64.7% of cats that are surgical candidates. Consequently, we evaluated the predictive ability of TBIL to identify BO in our population and determined the optimal cut‐off to discriminate between cats with and without BO to be ≥3.86 mg/dL, which had a sensitivity of 94.1% and a specificity of 82.4% to identify cats with BO. Consequently, to decrease the false negative rate of cats with BO, we propose lowering the previously suggested bilirubin cut‐off used to select cats for which abdominal imaging is critical to identify individuals requiring hepatobiliary surgery from ≥5.85 to ≥3.86 mg/dL. Although we acknowledge that applying this cut‐off will result in some cats being falsely considered surgical candidates instead of candidates requiring medical intervention (considering the decreased specificity at 82.4%), the possible life‐threatening nature of BO necessitates a more cautious approach to management whereby referral for abdominal imaging is needed. This finding hopefully will assist veterinarians in their decision‐making when presented with a cat with hyperbilirubinemia, as part of a thorough clinical assessment and consideration of other variables such as clinical history and physical examination findings.

Identifying surgical candidates is important to improve patient outcome. A recent prospective study (1980‐2019) evaluating 168 cats with hepatobiliary disease at a single referral center showed that individuals with BO (eg, because of cholelithiasis) treated by cholecystectomy had significantly longer survival times, older ages at death, and an overall better prognosis than cats with nonsurgical hepatobiliary diseases (eg, suppurative cholangitis‐cholangiohepatitis).^12^ Although less positive, similar research from another referral population evaluating the management of extrahepatic biliary tract disease in cats indicated that surgical management of BO carries a fair to guarded prognosis.^9^ Nevertheless, these 2 recent studies contrast with the results of an older study describing relatively high mortality and morbidity in cats with extrahepatic biliary tract surgery.^13^ In our study, >70% of cats (12/17) in which surgical management for BO was considered or recommended did not undergo surgery, either because the intervention itself was declined by the client or discouraged by the treating veterinarian. The reason why surgery often was recommended or at least considered was not always clear in the cat's medical records. Even though a better outcome compared to what has been historically considered seems likely based on the abovementioned studies,^9^, ^12^ it is possible that today veterinarians are still reluctant to recommend surgery, possibly because of an impression that hepatobiliary surgery for BO is commonly associated with poor outcome. More studies are needed to evaluate the morbidity and mortality associated with hepatobiliary surgery in cats.

Aside from bilirubin concentrations, our multivariable logistic regression modeling showed that as age increased, the odds of BO also increased significantly. Therefore, considering both age and TBIL in cats with hyperbilirubinemia is useful when assessing the likelihood of BO. This finding is interesting and suggests that conditions associated with BO might be more prevalent in older cats whereas, as already known, inflammatory conditions such as FIP^14^, ^15^ and nonsuppurative cholangitis‐cholangiohepatitis^16^, ^17^, ^18^ (as causes of hyperbilirubinemia) are more prevalent in younger animals. In people, obstructive jaundice is a common indication for abdominal surgery in the elderly,^19^ although it also can affect patients of any age, including neonates.^20^ Approximately one‐third of elderly patients with obstructive jaundice have malignant hepatobiliary neoplasia (also known as malignant jaundice).^19^ In people with obstructive jaundice of benign origin (nonneoplastic), elective hepatobiliary surgery is recommended shortly after the onset of the first symptoms of biliary tract disease.^19^ In our study, approximately 12% of cats with biliary disease had a mass identified on abdominal imaging, either within the biliary tree itself or affecting the duodenal papilla; BO was confirmed in all of them but we could not determine the malignancy of these masses. The average age for the cats with a mass affecting the biliary tree was 10 years old.

Our study had some limitations, mainly pertaining to its retrospective design. Although all medical records were systematically reviewed and a diagnosis was thoughtfully assigned to each case by the authors (in view of the documented diagnosis by the treating veterinarian and patient's medical records), there were a substantial number of cases in which the diagnosis was only tentative and a definitive diagnosis was not confirmed for several reasons, from clients declining further diagnostic testing to death or euthanasia during investigation. Likewise, the presence or absence of BO was assigned to each cat by the authors. The decision was based on thorough review of clinical records and imaging reports, but no standardized definition of BO was established for inclusion into this group. A BO also could have been missed in cats that did not undergo abdominal imaging, although appropriate diagnostic imaging was performed in the majority of cases (185/216). It is also possible that if further investigations had been performed, a cause for hyperbilirubinemia could have been found for the 80 cases in which the cause of hyperbilirubinemia remained uncertain, such as in cats with multiple trauma. In people, jaundice is described in trauma patients,^21^, ^22^ the main causes including bilirubin overload caused by breakdown of transfused or extravasated blood, hepatic dysfunction resulting from sepsis or infection, and initial shock and systemic hypotension after trauma. Not all cats with history of trauma in our study underwent complete abdominal imaging and, when done, subtle hepatobiliary changes could have been overlooked or not reported. Furthermore, because variations in the literature exist, classification of hyperbilirubinemia based on its origin (prehepatic, hepatic, posthepatic) was made at the authors' discretion, particularly when classifying systemic diseases such as FIP and sepsis, or disease processes affecting both the liver and biliary tree (eg, cholangiohepatitis). Nevertheless, even if other principles to classify hyperbilirubinemia causes into prehepatic, hepatic or posthepatic causes had been applied, doing so would not likely have changed the main study findings.

Because the study utilized a referral population to evaluate TBIL cut‐offs, the results might not be entirely representative for cats with hyperbilirubinemia presented to a primary care practice. However, we believe that the suggested cut‐offs still are valid and useful to assist primary care practice‐based veterinarians in their clinical decision‐making, such as the need for emergency referral for advanced imaging and potential surgery. Further research that addresses this limitation is required to evaluate TBIL cut‐offs for cats with hyperbilirubinemia in a general practice population.

Furthermore, even though all methods used to measure TBIL were validated using feline serum, it is possible that some bilirubin concentrations were artifactually increased by hemolysis^23^, ^24^ or lipemia,^25^ or both, which mainly would impact cats with very mild (and hence questionable) hyperbilirubinemia. Prospective evaluation of serum sample quality to evaluate for hemolysis and lipemia would have helped minimize inaccurate results, but doing so was not possible because of the retrospective nature of the study.

In conclusion, as part of a thorough clinical assessment, hyperbilirubinemia of variable severity has the potential to discriminate between cats that have BO and those that do not, and that may require emergency hepatobiliary surgery. We propose lowering the previously suggested bilirubin cut‐offs to select cats where abdominal imaging is considered critical to identify individuals requiring surgery, from ≥5.85 to ≥3.86 mg/dL (≥100 to ≥66 μmol/L), to avoid missing or delaying a diagnosis of BO. Additionally, the odds of BO increase with age. Therefore, considering both the age of the cat and severity of hyperbilirubinemia seems useful when assessing for the likelihood of BO.

## CONFLICT OF INTEREST DECLARATION

Authors declare no conflict of interest.

## OFF‐LABEL ANTIMICROBIAL DECLARATION

Authors declare no off‐label use of antimicrobials.

## INSTITUTIONAL ANIMAL CARE AND USE COMMITTEE (IACUC) OR OTHER APPROVAL DECLARATION

Approved by the CVS Group Ethics Committee (#CVS‐2022‐018).

## HUMAN ETHICS APPROVAL DECLARATION

Authors declare human ethics approval was not needed for this study.
